# Enhancing Banana Flour Quality through Physical Modifications and Its Application in Gluten-Free Chips Product

**DOI:** 10.3390/foods13040593

**Published:** 2024-02-16

**Authors:** Kannika Kunyanee, Tai Van Ngo, Sandra Kusumawardani, Naphatrapi Luangsakul

**Affiliations:** Department of Food Science, School of Food Industry, King Mongkut’s Institute of Technology Ladkrabang, Bangkok 10520, Thailand; kannika.ku@kmitl.ac.th (K.K.);

**Keywords:** dual-modifications, annealing, heat-moisture, banana flour, chip products

## Abstract

The objective of this study was to analyze the effects of different single or dual physical treatments, including pre-gelatinization (PBF), annealing (ANN), PBF+ANN, and ANN+PBF, on banana flour’s characteristics and its application in gluten-free chip production. The study involved determining the color, swelling capacity, solubility, oil absorption index, and pasting properties of both the native and modified banana flour samples. The results showed a significant change in color, particularly in the pre-gelatinized samples. There was a noticeable decrease in the values of the pasting parameters in the modified samples. PBF samples exhibited a remarkable reduction in the breakdown value compared to the native and ANN treated samples. Furthermore, PBF-treated banana flour displayed higher oil absorption and swelling power than the other samples, along with lower solubility in the PBF-treated sample. These characteristics appear to be responsible for enabling the pre-gelatinized sample to form the dough required for producing banana chips, resulting in distinct texture profiles. Finally, our research emphasizes the useful application of modified banana flour in the food industry and emphasizes how crucial it is to choose the right modification method to achieve the desired effects on the product.

## 1. Introduction

Bananas can be processed into various forms, including puree, juice, and flour, all of which offer extended shelf lives and useful applications [1]. To address these challenges, bananas can be transformed into semi-finished products, such as starch or flour, which provide advantages such as ease of use and longer shelf life compared to fresh bananas. Banana flour has been utilized in a variety of products, including pasta, bread, cakes, crackers, and cookies [2,3,4,5]. Furthermore, unripe banana flour, also known as green banana flour, is a rich source of starch, comprising resistant starch (RS) and dietary fiber, with potential health benefits when consumed. It contains antioxidant polyphenols with potent disease-fighting capabilities. Researchers are actively exploring new applications for banana flour in various food products [6]. Despite its promise, the native form faces limitations in the food industry, such as low heat stability and a tendency to retrograde during storage. However, realizing these benefits requires the modification of banana flour (both chemically and physically) to enhance its characteristics, such as improving thermal stability and adjusting its gel-forming properties to reduce stickiness. These modifications primarily operate at the molecular level of banana flour, known as its starch, with minimal impact on the external appearance of the starch granules while also promoting some health benefits. For instance, esterified unripe banana flour with citric acid has been used to develop cookies with low digestible starch and a low *p*-glycemic index [2]. Additionally, heat and moisture treatment modifications in banana flour have significantly altered its pasting properties and led to the highest Slow Digestible Starch (SDS) content [7]. Biscuits composed of heat-moisture-treated banana flour exhibited reduced blood glucose response, glycemic index, and glycemic load owing to their content of resistant starch and slowly digestible starch [8]. Incorporating pregelatinized banana flour into the instant porridge also enhanced its dietary fiber content, resistant starch (RS) content, and antioxidant capacity [9]. However, further exploration of the physical modification of starch is needed, as it can preserve natural properties and enhance versatility in application.

Some physical treatments show great potential in food modification, given their safety, environmental friendliness, cost-effectiveness, and the absence of chemical waste generation, all of which enhance product quality. One of these physical modification techniques is annealing treatment (ANN), which involves heating starch at temperatures below the initial gelatinization temperature but above the transition temperature under excess moisture content (>40%). Annealing has garnered significant attention due to its chemical safety, low cost, and environmental friendliness. It can greatly modify the functional properties of starch without disrupting the starch granular structure [10,11]. Notable changes in the functional properties of starch following annealing treatment include an increased gelatinization temperature as well as alterations in swelling power, starch solubility, amylose leaching, and pasting properties [12,13]. Various methods are available to create gluten-free products and enhance their texture, and one approach involves using pre-gelatinized starch in place of natural starch. Pre-gelatinized starch is utilized to improve the physical properties, density, and stability of colloid systems, thus enhancing their functionality and changeability for use in food products. As the food industry grapples with increasing demand for flour, banana flour emerges as a potential alternative to traditional sources, indicating its potential usefulness in various food applications. Single modification techniques have been applied and have altered some functional properties of the starch. However, it might not consistently provide all desired functionalities or properties in starch, constraining its application. Therefore, conducting dual modifications involving annealing and pregelatinization would offer new insights into improving the functional properties of banana flour, which would more applicable in the food industry. However, the sequence of modifications implemented will result in different effects on the properties of starch [14].

Therefore, the study aimed to investigate the effects of dual modifications, involving pre-gelatinization and annealing, on the physical properties, swelling power, solubility, oil absorption, and pasting properties of banana flour. The study also assessed its potential suitability for use in banana chip products.

## 2. Materials and Methods

### 2.1. Materials

Banana flour was purchased from a supermarket (Win gardens, Supahan Buri, Thailand). It had the following composition: 1.31% protein, 0.01% fat, 0.07% ash, which were analyzed using the described method of AOAC (2005). The amylose content (19.14%) of banana flour was determined by an iodine colorimetric method described by Fang et al. (2020) [15]. Other ingredients included: rice flour (Erawan^®^, Nakhon Pathom, Thailand), corn starch (Lilly^®^, Bangkok, Thailand), maltodextrin (Zhucheng Dongxiao Biotechnology, Weifang, China), monoglyceride (KC^®^, Chiang Mai, Thailand), shortening (Zest^®^, Samut Prakan, Thailand), and guar gum (KC^®^, Chiang Mai, Thailand). 

### 2.2. Modification of Banana Flour

#### 2.2.1. Preparation of Pregelatinized Banana Flour (PBF)

Banana flour modification using the pre-gelatinization method followed the method described by Waliszewski et al. [16] with slight modifications. The banana flour was mixed with distilled water to achieve a concentration of 65% (*w*/*v*). The slurry was transferred into an aluminum tray (30 cm × 30 cm × 3 cm) with a thickness of 2 cm and heated using an electric steamer (VC100630, Tefal, Créteil, France) with boiling water for 15 min. The gelatinized banana flour was then dried in a tray dryer at 50 °C overnight. The dried banana flour was ground into a fine powder and then passed through a sieve with a mesh size of 100 for further analysis. 

#### 2.2.2. Preparation of Annealed Banana Flour (ANN)

Banana flour underwent a treatment involving an excess of water, following a ratio of 1:2 (flour/water, *w*/*v*). A total of 300 g of banana flour was mixed with 600 mL of distilled water in a 1 L glass container, which was subsequently sealed with aluminum foil. The prepared sample was then subjected to incubation (MIR-23, Osaka, Japan) at a temperature of 55 °C for 6 h. Upon completion of the incubation period, the banana flour suspension was then centrifuged at 1000× *g* for 15 min, followed by a single washing with distilled water. The suspension was subjected to oven drying at a temperature of 50 °C overnight. The dried banana flour was finely ground into a powder and passed through a 100-mesh sieve for further analysis.

#### 2.2.3. Dual Modifications Using Sequential Treatments

To achieve dual modifications of banana flour, two sets of treatments were conducted: pre-gelatinization and annealing treatments (PBF+ANN) and annealing treatments followed by pre-gelatinization (ANN+PBF). First, the banana flour from Section 2.2.1, as described in the gelatinization method, underwent further modification through the ANN treatment explained in Section 2.2.2. Similarly, the banana flour treated with the ANN treatment from Section 2.2.2 was subsequently modified using pre-gelatinization, following the guidelines outlined in Section 2.2.1. 

### 2.3. Color of Modified Banana Flour

The color of banana powder samples was measured using a Hunter Lab scan colorimeter (Color Quest XE, Reston, VA, USA) equipped with a D65 illuminant and utilizing the CIE Lab* system. The color values were expressed as L* (whiteness/darkness), a* (redness/greenness), and b* (yellowness/blueness). Additionally, the Chroma (C*) and hue angle (h°) were calculated using Equations (1) and (2) below:

(1) $$C^{*}=\sqrt{{\left(a^{*}\right)}^{2}+{\left(b^{*}\right)}^{2}}$$

(2) $$h^{o}=\tan^{-1}\left(\frac{a^{*}}{b^{*}}\right)$$

### 2.4. Swelling Capacity and Solubility of Modified Banana Flour

The swelling power and solubility index of banana flour samples were determined following the method described by Hedayati et al. [17], with minor modifications. An amount of 0.1 g of banana flour was accurately weighed and mixed with 10 mL of distilled water. The mixture was immediately stirred using a voltage mixer for 30 s. The suspension was heated at 35 °C for 30 min with continuous mixing. After the heating process, the suspension was centrifuged at 4500 rpm for 20 min. The supernatants were carefully transferred into aluminum cans and then dried in a hot air oven at 105 °C until a constant weight was achieved. The swelling power and solubility index were calculated using the following Equations (3) and (4):

(3) $$Solubility\;\left(S\right)\left(\%\right)=\frac{dried\;supernatant\;weight\;(g)}{weight\;of\;flour\;(g)}\times 100$$

(4) $$Swelling\;power\;(\%)=\frac{residue\;weight\;(g)}{weight\;of\;flour}\times (100-S)$$

### 2.5. Oil Absorption of Modified Banana Flour

The oil absorption analysis of banana flour samples was conducted following the method of Bhosale and Singhal [18], with slight modifications. An amount of 0.5 g of banana flour sample was combined with 5 g of palm oil (Morakot, Thailand) for 30 s using a vortex mixer. After that, the mixture was allowed to stand at room temperature for 30 min and then subjected to centrifugation at 2000 rpm for 25 min. Subsequently, the supernatant oil was carefully decanted, and the amount of absorbed oil was calculated using the equation below (Equation (5)).

(5) $$Oil\;absorption\;\left(\%\right)=\frac{Weigh\;of\;a\;sample\;after\;centrifugation\;\left(g\right)-Weigh\;of\;a\;sample\;(g)}{Weigh\;of\;a\;sample\;(g)\times \left[100-moisture\;content\;\left(\%\right)\right]}$$

### 2.6. Pasting Properties of Modified Banana Flour

The pasting properties of the samples were determined according to the method described by Agyepong and Barimah [19], utilizing a Brabender Visco-amylograph (Visco-E, Brabender^®^ OHG, Duisberg, Germany). To determine the moisture content of each banana flour sample, an electronic moisture meter (MJ33, Mettler Toledo, Zurich, Switzerland) was employed. The moisture content value of a sample was then inputted into the Brabender Viscograph software (Version 4.2), which automatically provided the required weight of the flour sample and the quantity of distilled water needed to create flour suspension with a final weight of 450 g. Subsequently, the suspension was placed in the measuring bowl of the instrument and heated at a rate of 1.5 °C/min through a thermos-regulator. The heating process involved increasing the temperature from 50 to 95 °C, followed by a 15 min. holding period at 95 °C. Subsequently, the suspension was cooled to 50 °C for 15 min.

### 2.7. Application of Modified Banana Flour in Chips Product

#### 2.7.1. Preparation of Banana Chips

Banana chips were prepared using the following formula: 82.5 g of banana flour (with/without modification), 10 g of rice flour, 7.5 g of corn starch, 1.5 g of maltodextrin, 1 g of monoglyceride, 13 g of shortening, 0.5 g of guar gum, and 115 g of water. All ingredients were mixed for 5 min at speed 3 to form the dough using a KitchenAid Professional mixer (KitchenAid, St. Joseph, MI, USA). The dough was rolled to a thickness of 1 mm and cut using a 5 cm diameter round plate to shape the chips. The chips were then dried using a hot air oven (TO-772, OTTO, Bangkok, Thailand) at 180 °C for 12 min. After drying, the banana chips were cooled to room temperature and stored in polyethylene bags at 4 °C for further analysis.

#### 2.7.2. Texture Analysis

Texture measurements were conducted using a Texture Analyzer (TA-XT2i, Stable Micro Systems Ltd., Warrington, UK), employing a crisp fracture rig (HDP/CFS) and a 6.35 mm ball probe (P/0.25S). The tests involved placing the sample on the rig and driving a 6.35 mm ball probe, attached to the crosshead, perpendicularly at a speed of 1 mm/s. Texture measurements were taken 30 min after the sample was removed from the baking oven. From the force-deformation curve, the following parameters were obtained: hardness and fracturability value.

### 2.8. Statistical Analysis

The data were reported as mean ± standard by triplicate measurement. The data were analyzed for analysis of variance (ANOVA), followed by comparison of mean using Duncan’s multiple range tests at the 5% significance level. Statistical analysis was performed using the SPSS 22.0 statistical software program (IBM SPSS, New York, NY, USA).

## 3. Results and Discussion

### 3.1. Visualization Characteristics of Modified Banana Flour

**Figure 1:**
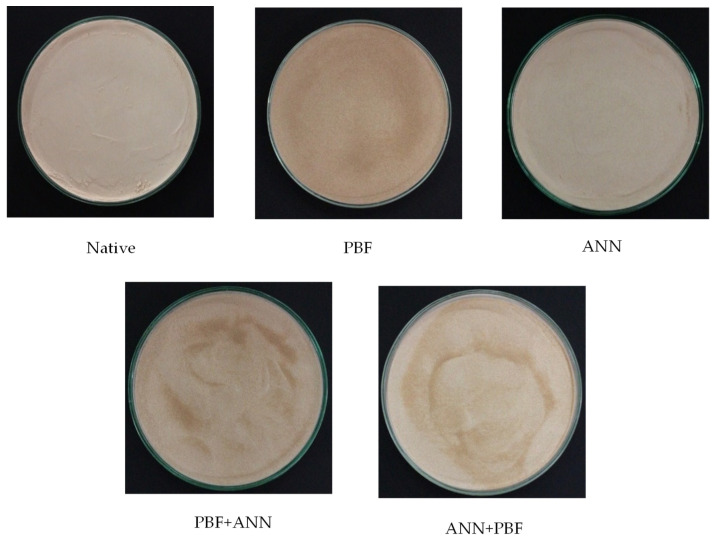
Characteristics of modified banana starch.

The appearance characteristics of unmodified banana flour (native) and modified banana starch by pre-gelatinization (PBF), annealing (ANN), and the combination of pre-gelatinization with annealing (PBF+ANN), and annealing followed by pre-gelatinization (ANN+PBF) were presented in Figure 1. Upon visual observation by the naked human eye, the modified flour revealed noticeable alterations in its appearance. Specifically, the color of the banana flour exhibited a shift towards darker, redder, and more yellow tones, aligning with a decrease in the lightness value shown in Table 1. The native color was brown. These changes might be attributed to the higher amount of oxidized phenol compounds formed [20,21]. However, each modification has different characteristics of appearance that are probably caused by the modification method [22]. Regarding the characteristics of banana flour under the different methods of modification, the most significant difference in flour color was observed in one of the banana flour samples. Specifically, the color of the flour subjected to either PBF treatment alone or PBF treatment in combination with annealing appeared darker. Pre-gelatinization is the process of exposing starch granules to heat and moisture, leading to their expansion and eventual rupture. This shows the starch’s internal components, which include starch molecules and trapped sugars, resulting in a higher concentration of sugars such as glucose and fructose within the exposed starch matrix [23]. The Maillard reaction, a complex chemical process that occurs when reducing sugars and amino acids interact with heat, is responsible for the browning observed in food products [24]. 

### 3.2. Color 

Color is one of the most important attributes for determining the quality of starch. In this study, we reported the measurement of the color characteristics of banana flour using color-instrumental parameters (L*, a*, b*, Chroma, and h*), which are presented in Table 1. L* indicates brightness (ranging from 0 to 100), while the parameters a* (indicating the red (+)/green (−) color attribute) and b* (indicating the yellow (+)/blue (−) color attribute) are essential in color analysis. Chroma reflects color intensity or saturation, and h* represents the hue angle for specifying color [25,26]. It can be observed that the brightness (L*) of native and modified flour, either through single-step PBF and ANN or dual-step combinations of PBF+ANN and ANN+PBF treatments, showed significant differences (*p* ≤ 0.05). The dual modification of PBF+ANN treatment of banana flour exhibited the lowest L* value (66.85), while the ANN treatment underwent minimal color alteration, possibly due to the mild treatment at a low temperature during the annealing process [26]. The reduction in brightness (L*) of modified flour may be attributed to color pigments such as flavonoids and carotenoids leaching out into the water during processing [27]. Pre-gelatinization treatment may reduce sugar concentration and, consequently, color development through the Maillard reaction. The Maillard reaction occurs during thermal treatment, where amino acids and reducing sugars induce non-enzymatic reactions that produce melanoidin, a high-molecular-weight compound resulting in a brownish color. Several physicochemical factors, including sugar concentration, types of amines and carbonyl groups, pH, temperature, heating time, and relative humidity, can influence the Maillard reaction [23,28,29]. PBF+ANN-treated banana flour exhibited the highest a* coordinate of chromaticity, indicating a more reddish tone, as shown in Figure 1. All banana flour had positive b* values, indicating a tendency toward a yellow color. The yellowness value (b*) of the native and modified banana flour differed significantly (*p* ≤ 0.05). It was observed that the dual modification of ANN+PBF treatment and PBF+ANN treatment resulted in a significant increase in yellowness compared to the native. The decrease in brightness and increase in redness and yellowness due to single and dual treatments of pre-gelatinization and annealing can be attributed to the Maillard reaction. The mobility of water and matrix molecules influenced by pre-gelatinization can affect the rate of the Maillard reaction. Additionally, higher temperatures during heating can accelerate brown coloration [29]. 

Furthermore, modified flour through PBF+ANN exhibited a higher C* value, indicating that this modification resulted in a more intense color compared to other treatments. However, the C* values for all the starches were relatively low. An increase in the C* value led to lower brightness (L*). All flour exhibited significantly different hue angle (*h*°) values, with hues leaning towards the yellow zone. The *h*° coordinate represents actual color, which is valuable for displaying the color appearance of food products [30]. The *h*° value of native and modified flours was significantly different (*p* ≤ 0.05). The lowest *h*° value was found in PBF+ANN-treated banana flour. However, all the banana flours exhibited relatively low *h*° values, supported by the increasing trend in a* values. This suggests a color range from yellow to slightly orange, possibly due to the presence of polyphenols, carotene, and other substances. Bananas are known to be rich in phenolic compounds, flavonoids, and catecholamines, making them good sources of antioxidants [31]. The color measurement of banana flour confirmed that modified banana flour had a darker color compared to the native, consistent with the values. The brightness (L*) and hue angle (*h*°) were reduced due to the PBF+ANN modification, as illustrated in Figure 1. Falade and Ayetigbo [32] reported that alterations in color values after starch modification by physical methods could affect the purification and separation of various heterogeneous materials, such as proteins, salts, sugars, and other elements.

### 3.3. Pasting Properties of Modified Banana Flour

The pasting properties of the native and single- or dual-physically treated banana flours are summarized in Table 2. During thermal processing, the required temperature led to the viscosity to increasing, which was called the pasting temperature [33,34]. The results showed a significant difference (*p* ≤ 0.05) in the effect of modification methods on pasting temperature. The pasting temperature of native banana flour was 80.20 °C, while PBF-treated banana flour exhibited the lowest pasting temperature of 64.55 °C, followed by the PBF+ANN sample at 69.20 °C and the ANN+PBF sample at 79.80 °C. The highest pasting temperature (81.25 ± 0.07 °C) was found in banana flour modified by the ANN treatment. The application of PBF treatment or PBF+ANN treatment significantly decreased peak viscosity, breakdown viscosity, final viscosity, and setback viscosity compared to the native. Due to the high temperature and moisture content during pregelatinizing, the starch structure was destroyed, and the breakage of covalent bonds inside starch molecules occurred, resulting in a change in the functional properties of starch [35]. ANN treated banana flour also reduced the peak viscosity, final viscosity, and setback value of banana flour. However, an increase in breakdown viscosity was found in the annealing treatment (103.5 ± 0.71 BU). Breakdown viscosity, the difference between peak and trough viscosity, is caused by granule disruption [33], and higher peak viscosity leads to higher breakdown viscosity. Furthermore, the annealing process might induce changes in both amylose and amylopectin, resulting in an amorphous structure and crystal structure. During annealing, the rearrangement of starch granules and interactions with other molecules, such as amylose, amylopectin, lipids, and proteins, led to a stronger starch structure, restricting swelling and reducing susceptibility to stirring action [36,37]. This was supported by the previous results that the peak viscosity of ANN treatment was higher at approximately 3-fold time than that of PBF treatment. The results also showed that the lowest peak viscosity, final viscosity, and setback viscosity were found in dual-treated samples, in which the ANN+PBF treatment had lowest value, followed by the PBF+ANN treatment. This upward trend of PBF+ANN treatment indicated that starch molecules have some degree of re-organization, and the hydrogen bonds within the amylopectin double helix undergo a change in the crystal structure to form a new stronger structure [38,39], while the pre-gelatinization led to an “order-to-disorder transition” starch structure that results in disruption easily [40].

### 3.4. Swelling Power and Solubility of Modified Banana Flour

The swelling power and solubility were observed in both native and modified starch (Table 3). Banana flour treated with PBF exhibited higher swelling power than the native, whereas ANN treatment resulted in a lower value. These differences in swelling power can be attributed to distinct interactions between the amorphous and crystalline starch chains [35]. The formation of weak hydrogen bonds during the pre-gelatinization process, coupled with the noted decrease in intermolecular forces, contributes to this variability. The leaching of amylose molecules and the release of hydroxyl groups, caused by the breakage of weak bonds due to heat, lead to a more freely swelling of starch [41,42]. PBF-treated banana flour exhibited the highest adsorption value at 2.99%, followed by the dual-treated samples (ANN+PBF and PBF+ANN). The lowest water swelling power was observed in the ANN sample at 1.56. This lower value can be attributed to the formation of a new structure that may restrict the swelling process, resulting in reduced swelling capacity [43]. Therefore, the combination of two of these methods could slightly reduce the swelling power of banana flour due to the annealing process. As explained by Gomes et al. [44], the annealing treatment enhanced the re-ordering and recombination of starch molecules in both the amorphous and crystalline regions, which is the main factor responsible for the reduction in swelling power. Moreover, Devi and Sit [45] showed that lower swelling was also found in annealed starches due to the perfection of crystallite starch molecules, which promotes the interaction of amylose and other components, which might hinder granule swelling. Combined with the rise in pasting temperature, it was confirmed that the annealing treatment facilitated the reorientation of amorphous amylose into a helical form, increasing the intermolecular interaction and modulating the interaction between crystallites and the amorphous matrix. Similar patterns have been reported by Eerlingen et al. [46] and Moorthy [47]. Whereas the heat from the pregelatinization treatment breaks the bonds in the starch granules, causing the granules to disrupt and allowing water to easily enter the hydroxyl group and form hydrogen bonds, resulting in a powder that absorbs water well [41,48].

The water solubility of both native and modified banana flours showed significant differences (*p* ≤ 0.05). The highest solubility was observed in the ANN treatment, while the native banana flour exhibited a water solubility of 10.46. Following this, the solubility decreased in the order of ANN+PBF, PBF, and PBF+ANN samples, respectively. The increase in the water solubility index of annealed starch in this study suggests that changes in the amorphous area and the disruption of hydrogen bonds between the amorphous and crystalline regions could occur, leading to the realignment of bonding forces within the starch granule. Consequently, this increases the binding force attraction and makes it more readily soluble in water [49,50]. The pregelatinization process could increase the starch solubility behavior [35]; however, a decrease in the soluble index was found in the pre-gelatinized sample compared to the native sample in this study. This may be because the excessive heating by the gelatinization process led to damage to starch structure and the loss of the free hydroxyl group, which resulted in a lower solubility index in the pregelatinized samples (single treatment and combined annealing process). The water absorption index and water solubility index of starch depend on the processing conditions and varieties of banana [35]. 

### 3.5. Oil Absorption Capacity of Modified Banana Flour

The oil absorption capacity exhibited a significant difference between the native and modified banana flours (*p* ≤ 0.05). The banana flour modified through pregelatinization displayed a higher oil absorption value compared to the native and other treated banana flours. This result suggests that banana flour modified by the pregelatinization method tends to have a higher oil absorption value. The modification process not only induces changes in the hydrophilic groups within the starch molecules but also enhances the density of hydrophobic groups on the surface of the starch structure, thereby improving the oil-binding capacity of the pregelatinized starch [47,51]. Another study also reported that annealing breadfruit starch did not lead to substantial changes in its oil absorption capacity [52]. The oil absorption of these flours was higher than that of the banana starch in the study of Thanyapanich et al. [53], which might be due to the contribution of non-starch as fiber to the capacity to absorb the oil. A study by Teli and Valia [54] showed that fiber from banana could be an oil sorbent. 

### 3.6. The Application of Modified Banana Flour in Chips Products

#### 3.6.1. Characteristics of Chip Products from Banana Flour

**Figure 2:**
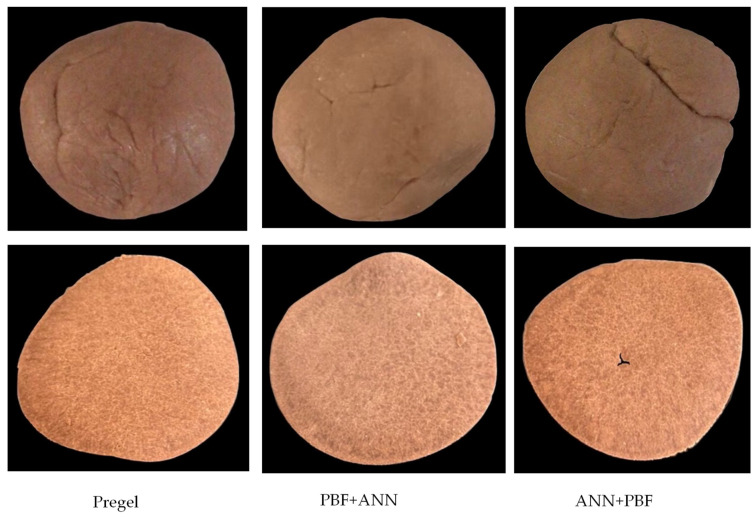
Appearance of dough balls and the appearance of chip sample made from banana flour.

Banana flour was applied to develop the chips products. However, the native and annealed modification (ANN) from banana flour cannot form a dough when mixed with water. Native starch is recognized to have low quality and many disadvantages, limiting its wide application in the food industry. It can form unattractive gels when the pastes are cooled, rendering them more cohesive. The gel can also easily undergo syneresis [55]. On the other hand, physical modification by annealing reduces water absorption capacity. The structure of the starch molecules becomes more compact, and the association of the starch polymer in the granule is strengthened, leading to increased roughness and cracked surfaces when applied to food products [56,57]. Therefore, the chips product could be molded by the flour that was modified by pregelatinization (PBF), the combined modification of pregelatinization and annealing (PBF+ANN), and the modified flour of annealing followed by pregelatinization (ANN+PBF). The appearance of the dough is shown in Figure 2. In terms of appearance, the dough treated with pre-gelatinization and annealing exhibited a smoother surface, likely due to the enhanced dispersion of pregelatinized starches and their increased water absorption. Additionally, the treatment with annealing contributed to improved viscosity profiles, fostering the enhancement of crystalline areas and interactions among starch chains [35,56,58]. Nevertheless, the appearance of a crispy snack made from banana flour modified by PBF treatment had the best texture and was less brittle. The brittleness of the chip also might be contributed by the fiber of banana flour; the poor bonding during dough preparation made the chip easy to crack. Flour with high water absorption can be mixed until homogenous, allowing it to be easily formed into sheets and shapes for crispy snack goods. This may be due to the pregelatinization process breaking the bonds of the starch granule structure. As a result, the pregelatinized starch can swell and bind well with water molecules. The pregelatinized process can improve dough consistency and reduce excessive dryness [59]. This is consistent with the water absorption effect shown in Table 3 for the application of crispy snack products. 

#### 3.6.2. Texture Profile

The texture profiles of chip samples prepared from both native and modified banana flour, including hardness and fracturability, are summarized in Table 4. Chips produced through the PBF treatment exhibited the highest hardness values compared to those from the PBF+ANN and ANN+PBF treatments. This difference can be attributed to starch gelatinization, which leads to the formation of a starch network structure, thereby enhancing the texture properties of the banana flour chips. The starch structure in these chips is maintained as a three-dimensional network interconnected by amylose-based crystallites, contributing to the product’s texture. In contrast, banana chips made through the PBF+ANN and ANN+PBF treatments showed a decrease in hardness values compared to PBF modification. This result was supported by the setback value, indicating that PBF treatment may disrupt some of the crystalline structures and amylose-amylose and/or amylose-amylopectin interactions. Consequently, the application of dual modifications to banana flour in chip products results in a softer texture, as illustrated in Table 4. 

Fracturability is a texture parameter that describes how a material responds to force, especially in the context of food and solid products. This indicated the material’s propensity to break or fracture under applied stress. A higher fracturability value indicates that the material is more prone to breaking or shattering under pressure, reflecting a brittle or fragile texture. In contrast, a lower fracturability value suggests that the material is less likely to break, signifying a more cohesive or flexible texture. The result showed that chips prepared from the PBF+ANN treatment had the lowest fracturability, indicating a texture that was less prone to breaking or fracturing, while the PBF treatment resulted in chips that were more fragile and likely to break when subjected to force. The chips subjected to ANN+PBF treatment displayed a fracturability similar to that of the PBF-treated chips, suggesting that annealing before pre-gelatinization had a limited impact on the chips’ tendency to fracture.

#### 3.6.3. Correlations between the Physicochemical Properties of Banana Flour and Banana Chip Quality

To recognize and control the quality of products made from banana flour requires an understanding of its properties, such as its pasting properties, its swelling power, its solubility, and its ability to absorb oil. These unique characteristics of the flour contribute to a variety of product attributes that affect customer choices about what to buy or consume. Furthermore, a product’s mouthfeel is greatly influenced by textural characteristics such as fracturability and/or hardness. As a result, the study intended to evaluate the relationship between the properties of banana flour and the characteristics of banana chips. The correlation among all parameters of modified banana flour, experimenting with single treatments of pre-gelatinization or annealing, and the combined dual treatment is represented in Table 5. Notably, the hardness of the texture exhibits strong positive correlations with both swelling power (SP) and oil absorption index (OAI) (R = 0.931 and R = 0.849, respectively). These findings are similar to those of Rani et al. (2019), who observed a comparable positive relationship between hardness and swelling power in their study involving multigrain flour-based noodle production. The observed changes in texture hardness seem to be intricately linked to the pre-gelatinization treatment’s impact. This treatment induces the leaching of amylose molecules and the consequent release of hydroxyl groups, precipitated by the thermal breakdown of weaker intermolecular bonds [2]. This process results in an increased capacity for starch to swell more expansively, leading to a structural alteration towards greater density and strength. Furthermore, a positive correlation is also shown between SP and OAI (R = 0.984), indicating that the modification not only induces changes in the hydrophilic groups within the starch molecules but also enhances the density of hydrophobic groups on the surface of the starch structure, thereby improving the oil binding capacity (Moorthy, 2002; Olayinka et al., 2008). However, hardness exhibits a negative relationship with pasting temperature (R = −0.886) but displays notably positive associations with other parameters, including peak viscosity (PV) (R = 0.872), final viscosity (FV) (R = 0.941), and set back (SBV) (R = 0.952). Fracturability as a parameter of material’s propensity to break under applied stress showed a positive correlation with setback value (SB) (R = 0.979), while breakdown viscosity exhibited a strongly negative correlation with fracturability and SB (R = −0.913 and −0.811, respectively). This finding was supported by a study conducted by Kaushal et al. (2012) on taro, rice, and pigeon pea flour. The breakdown viscosity is linked to how starch granules disintegrate or maintain paste stability during the holding time in a viscosity test. Therefore, when breakdown increases, solubility and fracturability tend to decrease. This could happen because an increase in breakdown viscosity indicates a quicker or more extensive breakdown of starch granules during heating. As a result, the starch might not retain its structural integrity or stability as effectively, leading to reduced solubility and decreased fracturability in the final product. Essentially, a higher breakdown viscosity might mean that the starch undergoes greater structural changes, impacting its solubility and resistance to fracturing. The interrelationships among the pasting viscosity parameters were noted. It was reported that pasting temperature (PT) exhibited a negative correlation with peak viscosity (PV), final viscosity (FV), and setback viscosity (SBV) with R values of −1.000, −0.991, and −0.985, respectively. Therefore, the characteristics of banana flour can be used as predictors of the qualities of chip products. Previous studies have emphasized the significance of banana flour qualities, such as its swelling power, solubility, and pasting ability, as key factors in predicting and controlling the quality of food items. These factors are important in determining customers’ decisions to buy or consume products [60].

## 4. Conclusions

The study investigated the effects of physical modifications on banana flour, notably observing significant changes in its appearance and color, particularly with the PBF+ANN treatment resulting in a darker color compared to other modifications, which was attributed to the Maillard reaction. The PBF treatment reduced pasting temperature and viscosity parameters, while the ANN treatment increased breakdown value. The PBF treatment increased the swelling power of the flour, while the ANN treatment decreased it. The dual modifications exhibited distinct trends, indicating a restructuring of the starch. Additionally, these modifications had effects on swelling power and solubility. Moreover, the annealing treatment, modified with other treatments, resulted in a significant change in solubility and oil absorption. Modified banana flour altered the banana chip texture, with PBF-modified flour generating a smooth texture in all gluten-free chip products made from banana flour. Overall, the research highlights the utility of modified banana flour in the food industry and underscores the importance of selecting the appropriate modification method to achieve desired product characteristics, contributing valuable insights into its potential applications, particularly in chip production.

## Figures and Tables

**Table 1:** The color of native banana flour and modified banana flour.

Samples	L*	a*	b*	Chroma	h*
Native	82.67 ± 0.09 ^a^	3.22 ± 0.03 ^d^	10.74 ± 0.13 ^b^	11.21 ± 0.13 ^b^	73.29 ± 0.05 ^a^
PBF	68.83 ± 0.08 ^d^	6.95 ± 0.01 ^a^	13.14 ± 0.09 ^a^	14.86 ± 0.08 ^a^	62.12 ± 0.16 ^d^
ANN	80.77 ± 0.08 ^b^	3.78 ± 0.12 ^c^	10.36 ± 0.08 ^c^	11.07 ± 0.08 ^b^	69.47 ± 0.04 ^b^
PBF+ANN	66.85 ± 0.45 ^e^	7.03 ± 0.03 ^a^	13.15 ± 0.11 ^a^	14.92 ± 0.09 ^a^	61.88 ± 0.29 ^d^
ANN+PBF	70.80 ± 0.06 ^c^	6.42 ± 0.01 ^b^	13.20 ± 0.08 ^a^	14.67 ± 0.08 ^a^	64.07 ± 0.09 ^c^

Mean ± S.D. followed by different letters within the same column are significantly different (*p* ≤ 0.05).

**Table 2:** Pasting properties of native and modified banana flour samples.

Samples	Pasting Temperature (°C)	Peak Viscosity (BU)	Breakdown Viscosity (BU)	Final Viscosity (BU)	Setback Viscosity (BU)
Native	80.20 ± 0.14 ^b^	763.50 ± 6.36 ^a^	84.50 ± 0.71 ^b^	861.50 ± 4.95 ^a^	182.50 ± 0.71 ^a^
PBF	64.55 ± 0.10 ^d^	193.50 ± 0.71 ^c^	3.50 ± 0.71 ^d^	292.00 ± 1.41 ^c^	101.00 ± 0.00 ^c^
ANN	81.25 ± 0.07 ^a^	601.00 ± 1.41 ^b^	103.50 ± 0.71 ^a^	621.00 ± 1.41 ^b^	125.50 ± 0.71 ^b^
PBF+ANN	69.20 ± 0.00 ^c^	159.50 ± 0.71 ^d^	19.00 ± 0.51 ^c^	215.00 ± 1.30 ^d^	74.50 ± 0.71 ^d^
ANN+PBF	79.80 ± 0.42 ^b^	71.00 ± 1.41 ^e^	10.00 ± 0.25 ^e^	111.00 ± 1.41 ^e^	43.00 ± 0.00 ^e^

Mean ± S.D. followed by different letters within the same column are significantly different (*p* ≤ 0.05). BU: Brabender Units.

**Table 3:** Swelling power, solubility, and oil absorption native and modified banana flour samples.

Samples	Swelling Power (g/g)	Solubility (%)	Oil Absorption (%)
Native	${1.73\pm 0.01\;}^{c}$	${10.46\pm 0.28\;}^{b}$	${2.22\pm 0.05}^{\;b}$
PBF	${2.99\pm 0.01\;}^{a}$	${9.10\pm 0.06\;}^{c}$	${2.42\pm 0.11\;}^{a}$
ANN	${1.56\pm 0.04\;}^{d}$	${10.95\pm 0.02\;}^{a}$	${2.07\pm 0.07\;}^{b}$
PBF+ANN	${2.66\pm 0.02\;}^{b}$	${8.09\pm 0.03}^{\;d}$	${2.15\pm 0.06\;}^{b}$
ANN+PBF	${2.70\pm 0.01\;}^{b}$	${9.36\pm 0.23\;}^{c}$	${2.23\pm 0.03\;}^{b}$

Mean ± S.D. followed by different letters within the same column are significantly different (*p* ≤ 0.05).

**Table 4:** Texture profiles of chips product from modified banana flour.

Samples	Hardness (g.)	Fracturability (mm.)
PBF	3976.70 ± 18.16 ^a^	1.33 ± 0.79 ^a^
PBF+ANN	2430.60 ± 13.53 ^b^	0.45 ± 0.30 ^b^
ANN+PBF	1860.70 ± 16.42 ^c^	1.32 ± 0.57 ^a^

Mean ± S.D. followed by different letters within the same column are significantly different (*p* ≤ 0.05).

**Table 5:** Correlation analysis.

	Hard.	Fract.	SP	SB	OAI	PT	PV	BD	FV	SBV	L*	a*	b*	C^o^	h*
Hard.	1.00														
Fract.	0.27	1.00													
SP	0.93	0.60	1.00												
SB	0.07	0.98	0.43	1.00											
OAI	0.85	0.74	0.98	0.58	1.00										
PT	−0.89	0.21	−0.66	0.40	−0.51	1.00									
PV	0.87	−0.24	0.63	−0.43	0.48	−1.00	1.00								
BD	−0.64	−0.91	−0.88	−0.81	−0.95	0.21	−0.18	1.00							
FV	0.94	−0.08	0.75	−0.28	0.62	−0.99	0.99	−0.34	1.00						
SBV	0.95	−0.04	0.77	−0.24	0.65	−0.99	0.98	−0.37	1.00	1.00					
L*	−0.26	0.86	0.11	0.95	0.29	0.68	−0.70	−0.58	−0.57	−0.54	1.00				
a*	0.62	−0.59	0.29	−0.74	0.11	−0.91	0.92	0.21	0.85	0.83	−0.92	1.00			
b*	−0.81	0.35	−0.54	0.53	−0.38	0.99	−0.99	0.06	−0.96	−0.95	0.78	−0.96	1.00		
C^o^	0.53	−0.68	0.18	−0.81	0.00	−0.86	0.88	0.32	0.78	0.76	−0.96	0.99	−0.93	1.00	
h*	−0.63	0.58	−0.31	0.73	−0.13	0.92	−0.93	−0.19	−0.86	−0.84	0.91	−1.00	0.97	−0.99	1.00

Note: Hard. is hardness, Fract. is fracturability, SP is swelling power, SB is solubility, OAI is oid absorption index, PT is pasting temperature, PV is peak viscosity, BD is breakdown, FV is final viscosity, SBV is setback value. Red represents positive correlation, blue represents negative correlation, and the daker of the color is better correlation.

## Data Availability

The original contributions presented in the study are included in the article, further inquiries can be directed to the corresponding author.
